# In vivo study to assess dosage of allogeneic pig retinal progenitor cells: Long‐term survival, engraftment, differentiation and safety

**DOI:** 10.1111/jcmm.17332

**Published:** 2022-04-28

**Authors:** Murilo Batista Abud, Petr Baranov, Sara Patel, Caroline A. Hicks, David Leonardo Cruvinel Isaac, Ricardo Noguera Louzada, Pierre Dromel, Deepti Singh, John Sinden, Marcos P. Ávila, Michael Young

**Affiliations:** ^1^ 67824 Federal University of Goias Goiania GO Brazil; ^2^ Schepens Eye Research Institute Massachusetts Eye and Ear Harvard Medical School Boston Massachusetts USA; ^3^ ReNeuron Pencoed UK; ^4^ Postgraduate Program in Surgical Science School of Medicine Universidade Federal do Rio de Janeiro Rio de Janeiro Brazil; ^5^ 2167 Massachusetts Institute of Technology Cambridge Massachusetts USA

**Keywords:** retina regeneration, retinal progenitor cells, retinitis pigmentosa, stem cell

## Abstract

Despite notable efforts and significant therapeutical advances, age‐related macular degeneration remains the single most common reason for vision loss. Retinal progenitor cells (RPCs) are considered promising candidates for cellular treatments that repair and restore vision. In this allogenic study, the phenotypic profile of pig and human RPCs derived using similar manufacturing processes is compared. The long‐term (12‐week) survival of green fluorescent protein‐pig retinal progenitor cells GFP‐pRPC after subretinal transplantation into normal miniature pig (mini‐pig) retina is investigated. Human eyes are both anatomically and physiologically mimicked by pig eyes, so the pig is an ideal model to show an equivalent way of delivering cells, immunological response and dosage. The phenotypic equivalency of porcine and clinically intended human RPCs was established. Thirty‐nine mini‐pigs are used in this study, and vehicle‐injected eyes and non‐injected eyes serve as controls. Six groups are given different dosages of pRPCs, and the cells are found to survive well in all groups. At 12 weeks, strong evidence of integration is indicated by the location of the grafted cells within the neuro‐retina, extension of processes to the plexiform layers and expression of key retinal markers such as recoverin, rhodopsin and synaptophysin. No immunosuppression is used, and no immune response is found in any of the groups. No pRPC‐related histopathology findings are reported in the major organs investigated. An initial dose of 250 k cells in 100 µl of buffer is established as an appropriate initial dose for future human clinical trials.

## 1 INTRODUCTION

In recent decades, tremendous interest has been gained in culturing stem cells for regenerative medicine due to the cells’ ability to differentiate into any desired lineage.^1^, ^2^ Insights into cellular behaviour, and exciting models for studying the signalling pathways that lead to disease formation, are discovered by researching stem cells. Understanding the mechanisms used by these cells to interact with a host enables disease models to be created for treatment purposes.^3^ Studying the developmental stages of any tissue or organ is an especially valuable tool that has been provided by stem cells. This tool is extremely critical for the central nervous system, as healthy tissues are rarely available for testing and exploratory studies.^4^ Similar promise has been shown by stem cells in retinal development and regeneration work.^5^, ^6^ The feasibility of differentiating these cells into 3D organoids that readily form different layers of the retina has been demonstrated using embryonic and induced pluripotent stem cells.^7^, ^8^, ^9^ However, the lack of a suitable model is a major challenge faced by translational steps. Although rodents are considered good models for preliminary studies, there is high species variability in rodent and human developmental biology and surgical anatomy. The effects of stem cells in animal models that negate the species variability and xenograft effect are critical to understand. In previous studies by us, pigs have been demonstrated as useful models, especially for retinal examination, as pig developmental biology and surgical anatomy mimics human retinas.^10^, ^11^, ^12^ Furthermore, the human retina is better analogously resembled by the genetic makeup of pig retinal cells than rodent retinal cells.

A previous study by us shows that pig RPCs (pRPCs), expanded for 3 passages in 20% oxygen, followed by 1–2 passages in 3% oxygen, survived for 4 weeks after subretinal implantation into normal domestic pig retina and differentiated to display photoreceptor markers such as recoverin and rhodopsin, as determined by immunohistochemistry (IHC).^11^, ^12^ The pRPCs used in the present study have been expanded from isolation under 3% oxygen conditions and cryopreserved at Passage 8.^12^ The manufacturing process for cell preparation is equivalent to the process used for the manufacture of human RPC (hRPC) Drug Product (DP) with the same reagents, timings and procedures used.

In this study, the phenotypic profile of pig and human RPCs derived using similar manufacturing processes is compared, and the long‐term (12‐week) survival of GFP‐pRPC after subretinal implantation into the normal mini‐pig retina is investigated. Mini‐pigs, as opposed to normal pigs, are needed for studies longer than 6 weeks due to animal size and housing limitations. The impacts of cell dosage (volume and cell concentration) on implants are also investigated. Based on the methods of previous allograft studies,^11^, ^12^ immunosuppression is not used in any group. Therefore, cell survival under non‐immunosuppressed conditions is assessed. As secondary objectives, the effects of escalating doses of implanted cells on engraftment are investigated, pRPCs are phenotyped, and the safety and biodistribution of allogeneic RPC therapy are assessed. Information on the development and verification of surgery is provided to guide the clinical protocol for delivering cells into the subretinal space.

The scientific justification for an allogeneic porcine study is based upon several vital facts. Firstly, evaluations of the route and means of delivery of cell therapies and cell dosage are instructed by large animals like pigs. In this case, the pig eye is well‐suited to model the human retina and subretinal space.^13^ Secondly, valuable data on cell survival, integration or differentiation would not be provided by a human‐pig xenograft study design due to immune rejection and cell–substrate and/or cell–cell miscommunication from species‐specific surface molecule differences.^14^ Thirdly, and perhaps of paramount importance, the present study design best represents, in a different species, an allogenic study design that is intended for the human population. Useful information on the many vital issues noted above, and on the survival of grafted RPCs under the non‐immunosuppression regimen, is uncovered.^15^

The equivalency, in phenotypic expression, of pRPCs and clinically intended hRPCs is importantly established by this allogenic study.

## 2 MATERIALS AND METHODS

### 2.1 Animals and duration of study

This study was conducted per the expected criteria of Brazil's Animal Welfare Act and the Association of Research in Vision and Ophthalmology (ARVO) guidelines and approved by the Institutional Animal Care and Use Committee (IACUC). The animals used in this study were outbred mini‐pigs of the BR1 strain and were purchased from Minipig Pesquisa e Desenvolvimento (Rua Bairro do Papagaio, S/N Papagaio, Campina do Monte Alegre, Brazil. Animals were implanted between 5 and 6 months old, an age range at which the retina and other CNS regions are fully mature and the eyes fully developed. Both castrated males (no testicles, animal IDs 01–09 and 20–40) and females (no ovaries and uteri, animal IDs 10–18 and 19–28) were included. A long‐term survival time of 12 weeks was chosen, as it was hypothesized to be of sufficient duration to 1) observe any possible immune response to the grafted cells and 2) allow the grafted cells adequate time to integrate and differentiate. Between 6 and 8 animals were contained by each group.

### 2.2 GFP donor

Pigs of the NT5 line that were transgenic for green fluorescent protein (GFP) were used as donor animals for progenitor cell isolation. All were obtained from Professor Randall Prather of the University of Missouri. These transgenic animals expressed GFP in all nucleated cells and were generated using a CMV promoter in a replication‐deficient retrovirus vector.^16^ The transgenic porcine zygotes and fertile pigs were obtained following a nuclear transfer from porcine fibroblasts modified to express the enhanced GFP version.

### 2.3 Tissue collection from GFP donor

A pregnant sow at 60 days of gestation was placed under terminal anaesthesia, and the uterine horns and foetuses were removed through an abdominal incision. That same day, the tissue was stored on ice and transported from Columbia, Missouri, to Boston, Massachusetts. The foetal retina porcine progenitors were collected using the previously described methods.^12^ After enzymatic digestion, the cell‐containing tissue homogenates were grown in fibronectin‐coated flasks.

The age of the donor cells at isolation was chosen to be analogous to the donor age of the human cells used in our clinical trial (ClinicalTrials.gov Identifier: NCT02464436). As the gestational time and rate of differentiation are distinct for both pigs and humans, we estimated the similar developmental stage through a review of the literature and a histologic examination of developing pig retina. The chosen time of isolation in both species was the peak of rod photoreceptor cell birth.

### 2.4 GFP‐pRPC tissue isolation and expansion to passage 8

GFP‐pRPCs were thawed from previous isolation (cell bank frozen at Passage 2 and plated on fibronectin‐coated flasks [Akron AK9715‐0005, lot 14103140664]) for expansion in UltraCULTURE™ Medium, supplemented by 1× l‐glutamine (Gibco), 20 ng/ml recombinant human EGF, and 10 ng/ml of recombinant human bFGF. Peprotech was >70% viable upon thaw. Cells were plated at a density of 20,000 cells/cm^2^ in T75 (Corning) for Passage 1 and from Passages 4 to 7 – to T500 (Nunc Delta). Flasks were kept in low oxygen incubator (3% O_2_, 5% CO_2_, 37°C, 100% humidity) for 48 h before passaging. Cells were collected using Trypzean and then treated with both DTI‐benzonase and centrifugation. Cells were re‐plated on new flasks with the same density. >95% of passaged cells were viable. On average, population size was doubled every 36 h. GFP‐pRPCs were frozen at Passage 8 in Ultra‐culture medium and supplemented by 10% DMSO as antifreeze. There were 5 × 10^6^ cells contained per vial.

### 2.5 pRPC for implantation

GFP‐pRPCs were isolated from developing retina and expanded in 3% oxygen using the same medium composition as hRPC (Ultra‐Culture medium, 10 ng/ml recombinant human EGF, 20 ng/ml recombinant human bFGF, L‐glutamine) on fibronectin‐coated flasks. Cells were expanded to Passage 8 in 3% O_2_, then frozen and shipped to the surgery site (Federal University of Goias), where cells were revived and cultured for 48 h in 20% O_2_ before formulation. For injection, cells were collected from flasks and formulated in HBSS‐NAC as a suspension of 8 × 10^4^ to 1.2 × 10^5^ cells/µl (for dosing Group F). Cells for dosing groups B to E (Table 1) were diluted in HBSS‐NAC from this concentration to give 2.5 × 10^3^ cells/µl (Group C), 5 × 10^3^ cells/µl (Group B), 1 × 10^4^ cells/µl (Group D) and 5 × 10^4^ cells/µl (Group E).

**TABLE 1:** Study design

Group	Agent	Volume, μl	Cell number, total dosage, in millions	Cell conc	Number of eyes	Number of pigs	Scheduled sacrifice time w = weeks h = hours
A	HBSS‐NAC	100	0	0	4	4(1F/3M)	1@3w, 3@12w
B	pRPC in HBSS‐NAC	50	0.25	5000/μl	7	7(4F/3M)	1@3w, 5@12w
C	pRPC in HBSS‐NAC	100	0.25	2500/μl	6	6(4F/2M)	1@3w, 5@12w
D	pRPC in HBSS‐NAC	100	1	10,000/μl	7	7(1F/6M)	1@24h 1@3w, 5@12w
E	pRPC in HBSS‐NAC	100	5	50,000/μl	8	8(4F/4M)	1@3w, 6@12w
F	pRPC in HBSS‐NAC	100	10	100,000/μl	7	7(3F/4M)	1@24h 1@3w, 5@12w

### 2.6 Surgery and implantation

Animals were treated according to the study design shown in Table 1. All animals were pre‐anaesthetized with intramuscular injections of 15 mg of midazolam and a cocktail consisting of 11.9 mg/ml of zolazepam, 11.9 mg/ml of tiletamine, 12.38 mg/ml of xylazine, 14.29 mg/ml of ketamine (Intervet) and 2.38 mg/ml of methadone. The pigs underwent endotracheal intubation and were artificially ventilated with 2%–3% isoflurane combined with oxygen. Stroke volume and respiratory frequency were maintained at 300 ml/stroke and 12/min, respectively. In each case, the left pupil was treated with topical drops consisting of a combination of 0.4% oxybuprocaine, 10% Metaoxedrin, 0.5% Mydriacyl, 1% atropine and 5% povidone‐iodine. At surgery, the central and posterior vitreous was removed together with the posterior hyaloid membrane using a 25G three‐port pars plana vitrectomy.^10^ GFP‐pRPC (cell suspension in HBSS‐NAC) was injected subretinally in the para‐foveal region, 4–5 mm superomedial to the optic nerve head, through a 38‐gauge needle connected to a Hamilton syringe. Arcade vessels were avoided. Injections were given at an approximate rate of 200 μl/min. A 10–25 μl air bubble was delivered at the end of the injection to seal the retinotomy.

### 2.7 Animals and groups

Thirty‐nine mini‐pigs were used in this study. The controls maintained were vehicle‐injected eyes and fellow non‐injected eyes. Animals were treated according to the design in Table 1. Treatments were randomized across treatment groups, operators and surgery days as much as possible (Table 1).

### 2.8 Immunosuppression

In a previous allogenic study, no significant improvement in cell survival was indicated in pigs that received intravitreal rapamycin.^11^ Therefore, no immunosuppression was administered in this study.

### 2.9 Other treatments administered

As a precautionary measure, two doses of antibiotics (PENCIVET^®^ PLUS PPU [MSD; 8.000 IU/kg]) were administered to recipients. The first dose was administered immediately post‐surgery, and the second dose was administered two days later.

### 2.10 Eye examination and animal monitoring

Following the subretinal implantation of cells, fundus and eye examinations were conducted on study days 1, 7, 21 and 90 to monitor retinal detachment, bleb resolution, inflammation, symptoms of infection and clarity of the lens.

Body weights were measured, and eye examinations were performed at the start, end and appropriate intervals of the study. Food intake, general health and distress and anxiety symptoms were checked daily by attending animal facility staff. The dates and natures of any adverse events that may have occurred were noted. Eye examinations were conducted weekly under general anaesthesia. If present, retinal detachments (bleb size) were noted.

### 2.11 Blood serum samples and measurement of IgG and IgM levels

Animals were sedated with ketamine (10 mg/kg), azaperone (0.5 mg/kg) and midazolam (0.5 mg/kg) for blood collection. Through a venous puncture in the leg, blood was collected in BD Vacutainer^®^ SST™ Tubes (BD Catalog # 367986) with clot activator and gel for serum separation. Tubes were then maintained at −80°C until analytical analysis. IgG and IgM levels were measured in blood serum using standard ELISA methods and kits. The blood sampling schedule is shown in Table 2.

**TABLE 2:** Blood sampling

Blood sampling day	Day 1	Day 7	Day 21	Day 28	Day 90
Animals sampled	All animals	All 3‐week and 12‐week animals	All 3‐week animals	All 12‐week animals	All 12‐week animals

### 2.12 Necropsy

Animals were sacrificed at either 24 h, 3 weeks or 12 weeks after implantation (Table 1) under the supervision of a certified veterinarian. An overdose of pentobarbital was delivered by intravenous injection, and deaths were confirmed by the attending veterinarian.

Eyes, and a minimum of 5 mm of the optic nerve, were enucleated within 5 min of sacrifice and fixed in cold Davidson's fixative or 4% paraformaldehyde (24 h). The implanted eyes were enucleated first and subsequently used to harvest ocular tissue. The following tissues and organs were then extracted: the brain, spleen, submandibular lymph nodes, thymus, heart, lungs, liver, pancreas and kidneys. Following gross examination, each tissue was dissected, trimmed and fixed in 10% neutral buffered formalin.

Blood samples were collected in standard blood tubes for serum analysis of IgG and IgM to assess possible immune reaction to the allogeneic pRPC treatment.

### 2.13 Histology

All tissues, including eyeballs, were processed to paraffin blocks and sectioned.

GFP IHC on ocular tissues was conducted using standard indirect fluorescence immunohistological methods. Haematoxylin and eosin (H&E) histopathology was also performed on eyes and major organs to obtain information on RPC safety.

### 2.14 Tissue sampling procedures for eyes and organs

#### 2.14.1 Eyes

Cell‐treated (left) eyeballs were bisected in the sagittal plane and embedded in two paraffin wax blocks. 5‐µm sections were collected on charged glass slides at 15‐µm intervals throughout Block 2. This region of interest (Block 2) was estimated based on observations of GFP‐positive staining in serial sections collected across a 4–6 mm region from Blocks 1 and 2 of the hemisected eyes taken at 24‐hour post‐implantation. If no GFP cells were detected in Block 2 of the hemisected injected eyes, serial sections were collected identically from Block 1 for analysis.

Control untreated (right) eyeballs were bisected in the sagittal plane and embedded in two paraffin wax blocks. 5‐µm sections were collected at 15‐µm intervals at depths of 300, 1800, 3300 and 4800 µm into Blocks 1 or 2, determined by the block location of the injection site and/or survival of pRPCs in the treated eye.

#### 2.14.2 Organs

5‐µm sections of the brain, lungs, heart, kidneys, liver, pancreas, thymus, submandibular lymph nodes and spleen were taken for H&E histopathology assessments. Additionally, 3 serial sections of all tissues were taken for GFP IHC to assess the biodistribution of pRPC to major organs and immune tissues.

#### 2.14.3 Immunohistochemistry (IHC)

Fluorescence IHC was used to detect GFP‐pRPC in host pig tissues (treated left eyes, untreated right eyes and organs, and immune tissues) using chicken anti‐GFP antibody (Abcam ab13970; 1:750) and anti‐rabbit Alexa 488 conjugated secondary antibody (Invitrogen, A11039; 1:300), followed by a Hoechst counterstain. The microscopic analysis for pRPC phenotype and proliferation was evaluated using dual‐label fluorescence IHC with the antibodies listed in Table 3.

**TABLE 3:** Antibodies used for the evaluation of injected pRPC and effect on host tissues

Antigen	Species	Description	Used for detection of	Supplier	Product code	Dilution
GFP	Chicken	Green fluorescent protein	Grafted cells	Abcam	ab13970	1:750
Ki67	Rabbit	Ki67	Proliferating cells	Abcam	ab15580	1:500
CD45	Rabbit	Leucocyte Common Antigen	Leucocytes	Abcam	ab10558	1:200
Recoverin	Rabbit	Recoverin	Cones	Millipore	AB5585	1:500
Rhodopsin	Mouse	G protein coupled receptor 1	Rods	Millipore	MABN15	1:500
Synaptophysin	Mouse	Synaptophysin	Fiber synapses	Serotec	MCA1307	1:25

#### 2.14.4 Assessment and quantification of distribution of GFP‐pRPC (surface area)

**FIGURE 1:**
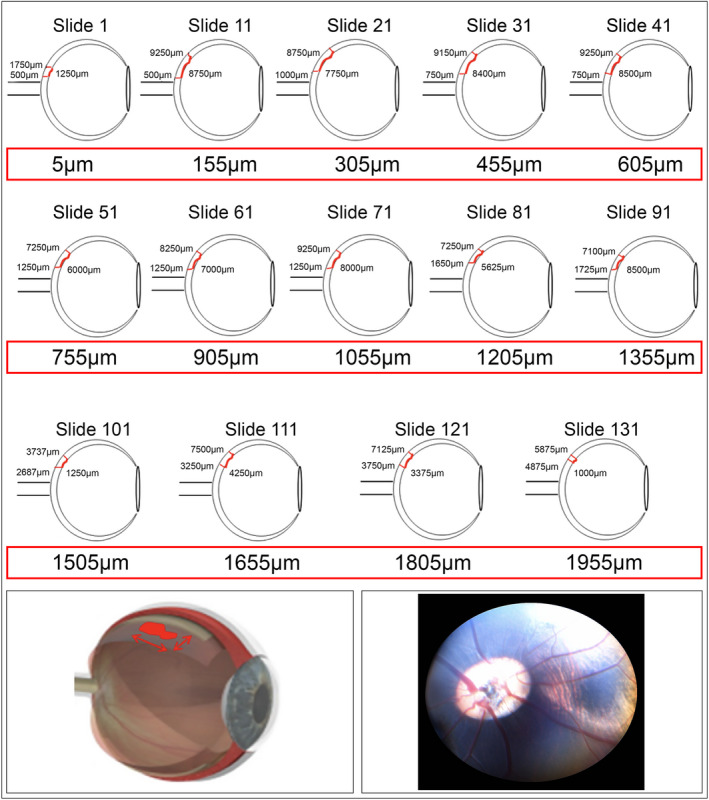
Example of cell graft distribution in retina and calculation of graft surface area in mm^2^ and pigmentary changes seen at retinography at 12 weeks

Microscopic assessments were performed on a Leica DMRB microscope equipped with epi‐fluorescence optics and Image ProPremier (Version 9.0) image analysis software. Stained sections were examined to determine the presence or absence of GFP‐positive cells. For every section demonstrating GFP staining, the cell graft was mapped. The details were either recorded on template illustrations of the eyeball or eyes taken at the 12‐week post‐treatment interval (see example in Figure 1).

Using a calibrated eyepiece graticule, the distance (length) over which GFP‐positive staining was observed was recorded for each section in a series. The mean length was determined by multiplying the distance over which GFP‐positive staining was observed to derive the total surface area coverage by graft expressed in square millimetres (mm^2^). The graft remaining in the subretinal space was contiguous. However, cells integrated into the outer nuclear layer (ONL) and inner nuclear layer (INL) were also identified.

#### 2.14.5 Assessment of proliferation potential of surviving pRPCs

The proliferation potential of surviving pRPCs was assessed using dual‐labelled IHC‐deploying GFP and Ki67 antibodies. The number of GFP‐positive cells and the number of co‐labelled GFP+Ki67+ cells were counted. The percentages of GFP‐positive cells co‐labelled with the Ki67 antibody were calculated to give a pRPC proliferation index.

#### 2.14.6 Assessment of host immune response in treated eyes

Sections from the left treated eyes were stained with an antibody against CD45 to assess local immune response. The number of CD45‐positive cells was counted, and averages were obtained for each dose group.

#### 2.14.7 Assessment of pRPC phenotype

Dual‐label fluorescence IHC methods were employed to investigate the differentiation of the surviving GFP‐positive cells. The markers used are listed in Table 3.

#### 2.14.8 Biodistribution analysis

Biodistribution analysis, using the GFP IHC methodology established for assessing cell survival in the treated eyes, was performed on sections serial to those evaluated for H&E histopathology. Sections of the brain, heart, kidneys, liver, lungs, pancreas, spleen, submandibular lymph node and thymus were processed for GFP IHC.

#### 2.14.9 Haematoxylin and eosin (H&E) histopathology

Tissue processing, sectioning and H&E histopathology were performed by an independent laboratory. Four sections per left and right eye, and sections of other tissues (brain, heart, kidneys, liver, lungs, pancreas, spleen, submandibular lymph node and thymus), were stained with H&E and examined by a veterinary pathologist.

## 3 RESULTS

### 3.1 Phenotype of porcine versus human RPCs

All major retinal and stemness markers were tested to compare the phenotype of porcine and human RPCs (Table 4). Cells used are mixed population of retinal cells and have not been purified for certain population. Hence, the cells are at different stages of differentiation and maturation as noted with PCR data. We believe these cells are good source if population of interest needs to be purified. Similarities in the genetic makeup of both pigs and humans were clearly indicated by the RT‐PCR comparison of porcine and human RPCs. Rhodopsin was found to be negative in both hRPCs and pPRCs. GFP‐pRPCs were found to be negative for MHC‐II in non‐stimulated conditions. RT‐PCR results were further concurred by ICC and flow cytometry analyses. Using phenotypic and genotypic markers, it was confirmed that the hRPC and pRPC expressions followed similar patterns.

**TABLE 4:** Comparison of expression of phenotype markers in undifferentiated human RPC and undifferentiated pig RPC by RT‐PCR, ICC and flow cytometry

Antigen (Ag)	Human RT‐PCR	Human ICC	Human flow cytometry	Pig RT‐PCR	Pig ICC	Pig flow cytometry
Crx	+	+	+	+	+	n/t
Nrl	+	+	+	+	+	n/t
Lhx2	n/t	n/t	n/t	+	+	n/t
Pax6	+	+	+	+	+	+
Klf4	+	+	+	+	+	+
Vimentin	+	+	+	+	+	+
Ki67	n/t	+	+	n/t	+	+
SSEA4	n/t	+	+	n/t	+	+
MAP2	+	+	+	+	+	+
GS	+	+	+	+	+	+
Recoverin	+	+	+	+	+	+
S‐Opsin	+	+	+	+	+	+
CD24‐conj	n/t	n/t	+	n/t	n/t	n/t
HLA‐ABC	n/t	+	+	n/t	n/t	n/t
Rhodopsin	−	−	−	−	−	−
Otx2	+	+	+	+	+	−
NeuroD1	n/t	+	+	n/t	+	+
CD73	n/t	n/t	+	n/t	n/t	n/t
CD38	n/t	n/t	−	n/t	n/t	−
CD133	n/t	n/t	−	n/t	n/t	−
Sox2	+	+	+	+	+	+
Pax6	+	+	+	+	+	+

### 3.2 Clinical observations after transplantation to the subretinal space

Clinical observations of eye and fundus examinations were considered uneventful for up to 12‐week post‐implantation. Surgical procedure‐related events included inflammation (redness), in rare cases in the treated eyes, and was resolved in 7 days. No inflammation was noted at the 3‐week or 12‐week intervals. Seven cases of sub‐capsular cataracts were observed, and 3 of them developed into full cataracts by 12 weeks. Cataract formation was considered procedure‐related. Subtle subretinal pigmentary changes were noted at the site of blebs in all 24 animals at the 3‐week and 12‐week intervals (Figure 1).

Through ophthalmoscopy of the fundus, it was revealed that all blebs had resolved within 7 days after treatment, and there was no retinal detachment observed in any animal. None of the clinical observation findings were dose‐related. Body weight increase was not affected by cell treatment. Food and water consumption was unaffected by treatment, and animals were found to gain weight steadily throughout the duration of the study.

**FIGURE 2:**
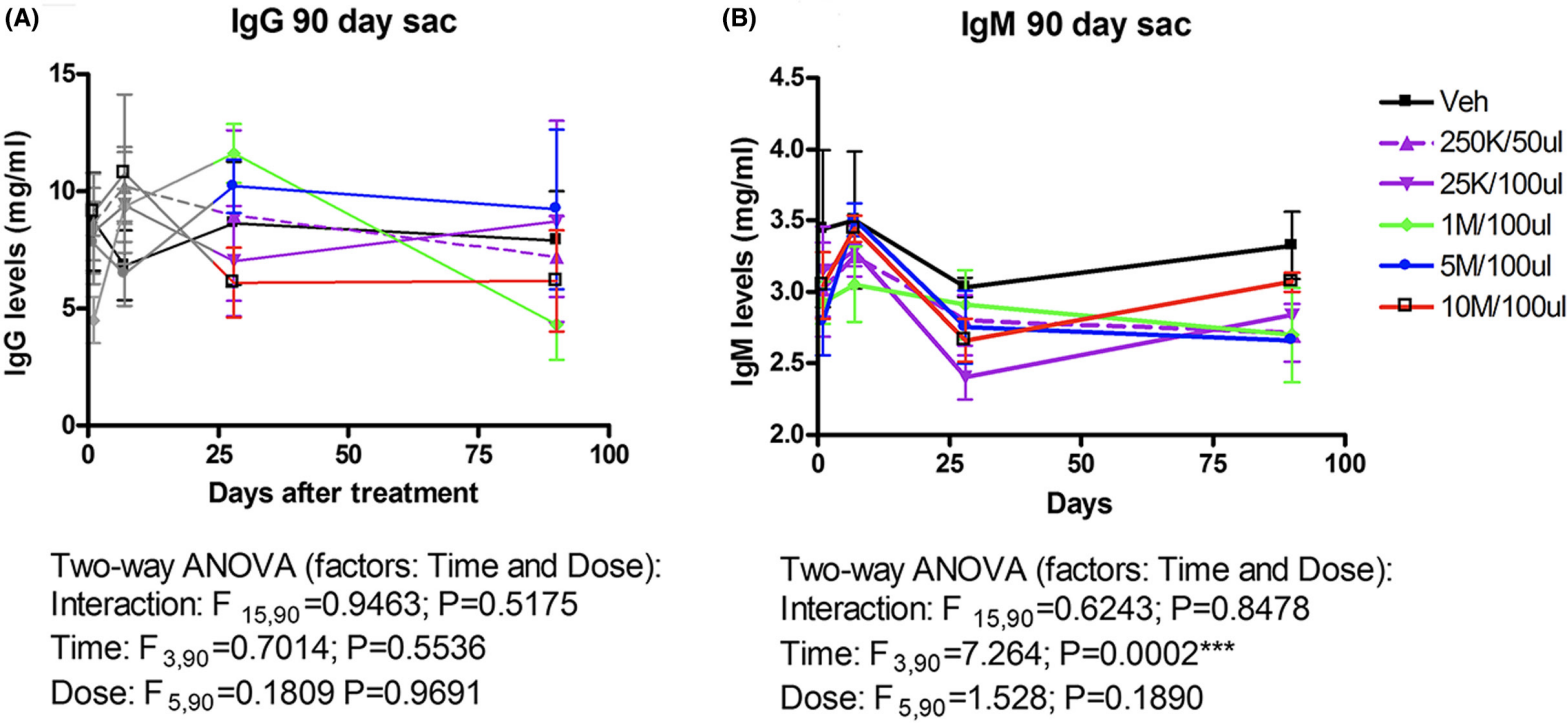
Blood serum IgG and IgM levels in animals sacrificed at 12‐week post‐treatment. It would be unlikely that a T‐cell response would be evoked by subretinal pRPC implantation. It is unlikely that a T‐cell response to elaborate memory and specific IgG

A blood analysis was performed, and serum IgG and IgM levels are shown in Figure 2. Two‐way ANOVA did not reveal a statistically significant interaction by dose and time (*F*_15,90_ = 0.9463; *p *= 0.5175) or by dose (*F*_5,90_ = 0.1809; *p *= 0.9691) or by time (*F*_3,90_ = 0.7014; *p *= 0.5536) on blood serum IgG levels after pRPCs were implanted in the subretinal space. Since no IgG developed after 28 days, it is unlikely that a T‐cell response (to elaborate memory and specific IgG) would be evoked by subretinal pRPC implantation.

Two‐way ANOVA did not reveal a statistically significant interaction between dose and time (*F*_15,90_ = 0.6243; *p *= 0.8478) or by dose (*F*_5,90_ = 1.528; *p *= 0.1890) on blood serum IgM levels after pRPCs were implanted in the subretinal space. Although a statistically significant effect was seen over time (*F*_3,90_ = 7.264; *p *= 0.0002), Bonferroni post‐test comparisons did not identify any differences between groups at different times. An apparent transient increase at 7 days, followed by a decrease at 28 days, was observed in blood serum IgM levels after subretinal implantation, which may be considered a non‐specific response to the surgical procedure.

### 3.3 pRPC survived and migrated to photoreceptor layer after transplanted to subretinal space

**FIGURE 3:**
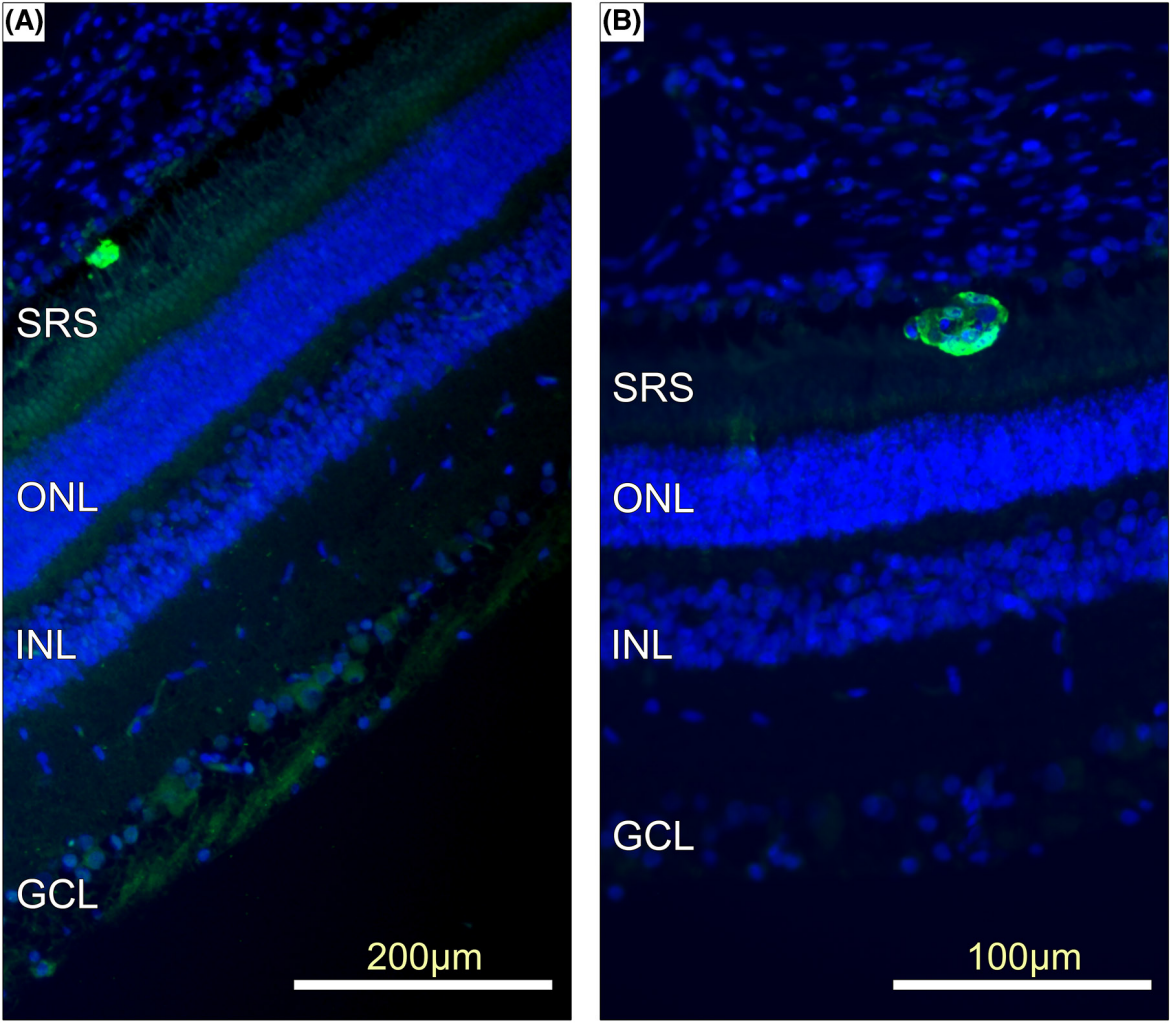
Photomicrographs of 5‐μm sections of retina taken at 24 h after treatment showing survival of implanted cells (green). Green: pRPC, Blue: Hoechst counterstain 24‐h images

**FIGURE 4:**
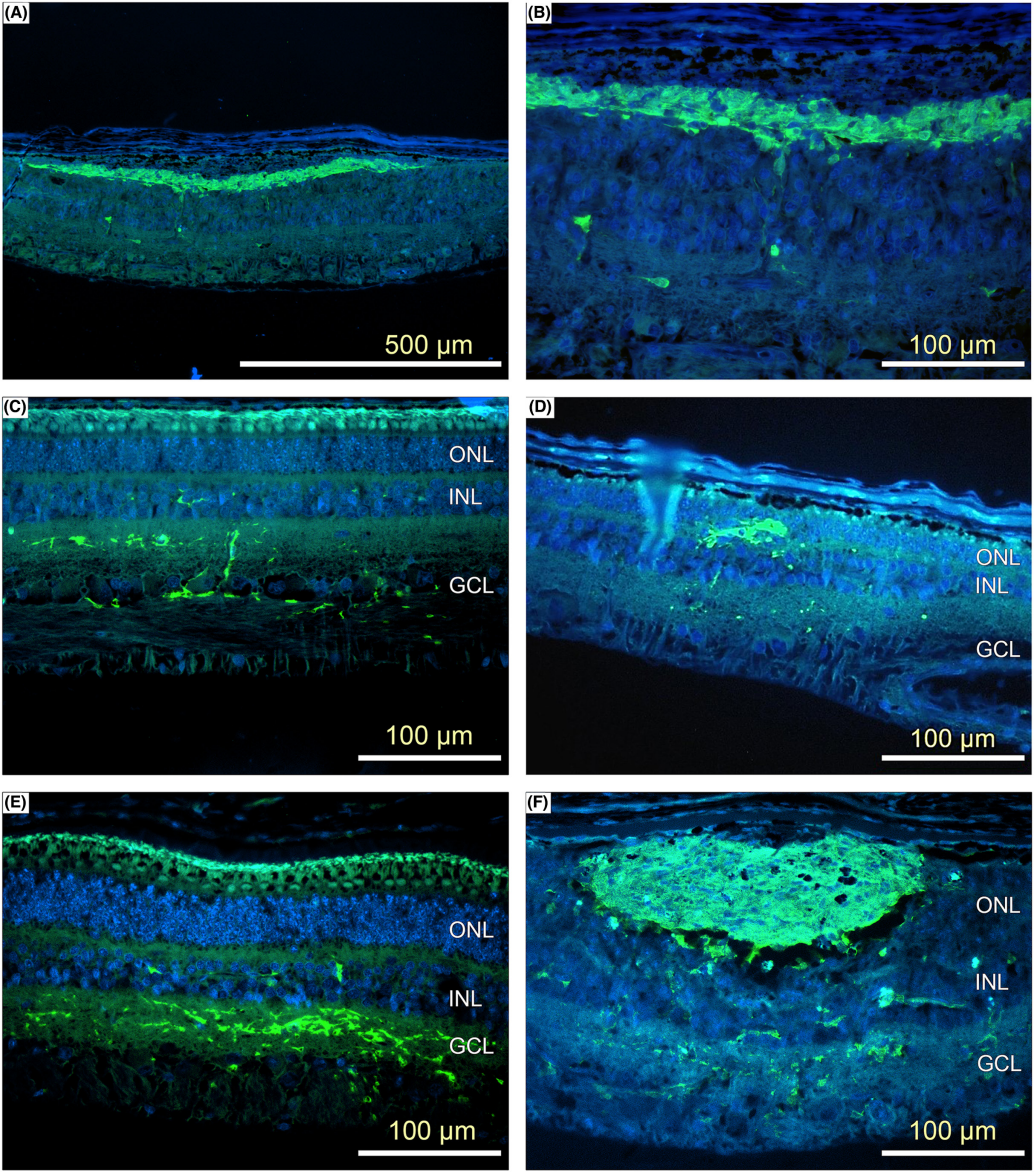
Photomicrographs of 5‐μm sections of retina taken at 3 weeks after treatment showing survival of implanted cells (green) at 3‐week post‐implantation (A–E). Green: pRPC; Blue: Hoechst counterstain and 24 h after implantation (F)

By examining the GFP‐stained histological sections, resolutions of subretinal blebs at the 24‐h and 3‐week post‐implantation intervals were compared. At 24 h, implanted cells were seen in the subretinal space (Figure 3) as either single entities or spheres of various sizes in both injected eyes. By 3 weeks, implanted cells were seen migrating into the neural retina (Figure 4A–E). Cells were seen in 80% (4/5) of the cell‐treated eyes. No positive GFP signal was detected in the vehicle‐treated eyes. The morphology of surviving cells seen at 24 h was distinct compared with those seen at 3 weeks. At 24 h, GFP‐positive signals were seen in the subretinal space as either single entities or as neurospheres of various sizes in the injected eyes (Figure 4F). The rounded morphology of the surviving cells seen at 24 h was different from the elongated cells seen at 3 weeks. A further difference was that the surviving GFP‐positive cells were juxtaposed to the photoreceptor layer at 3 weeks compared with the random positions in the subretinal space at 24 h. No GFP‐positive cells were noted in the untreated right eyes. No macrophage responses were reported, except in a single case where the injection cannula had penetrated the sclera.

### 3.4 GFP immunohistochemistry at 12‐week post‐treatment

**FIGURE 5:**
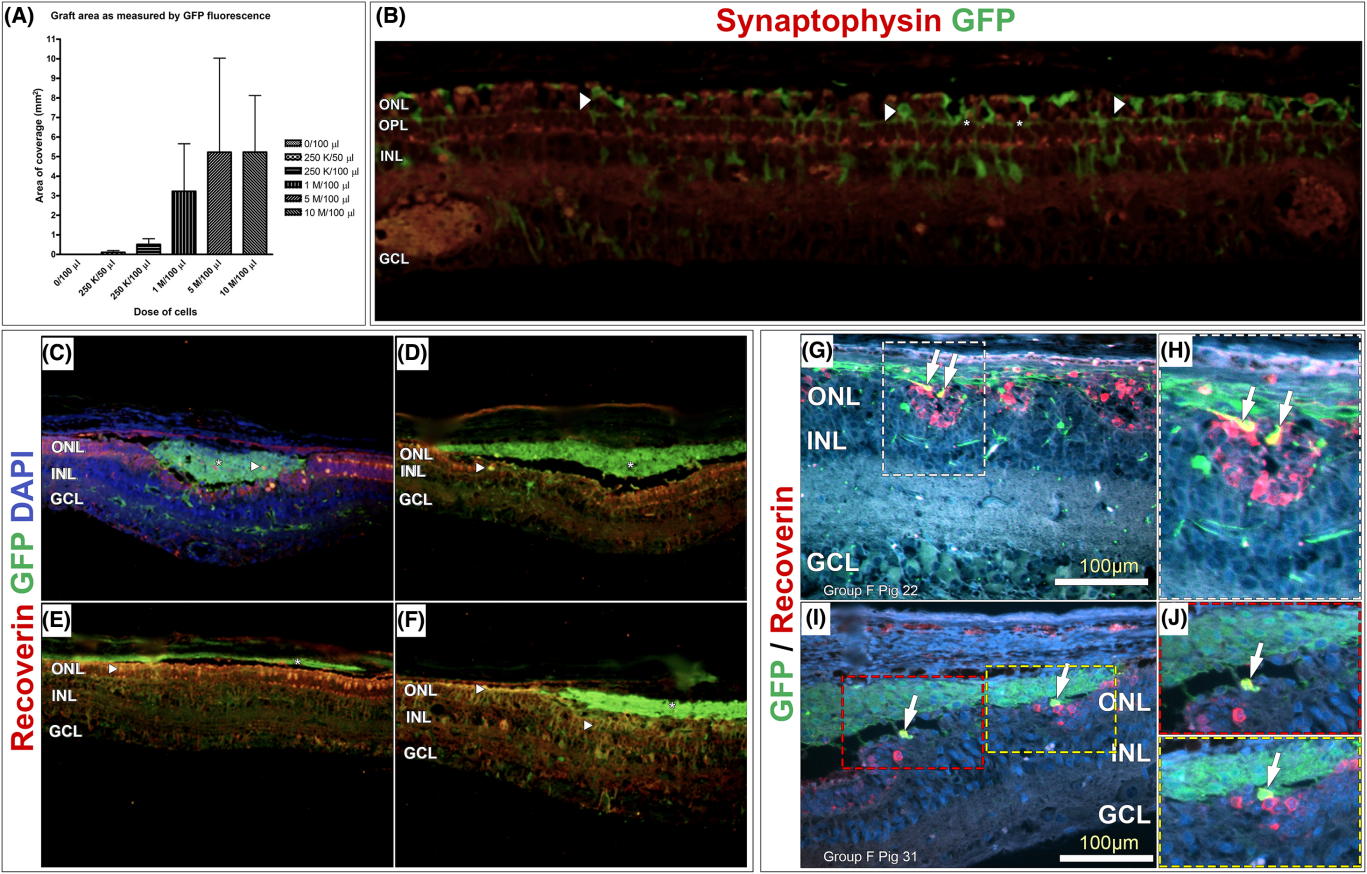
Bar graph showing the effect of dose on pRPC engraftment (A). Photomicrograph of 5‐μm section of retina taken at 12 weeks after treatment showing processes of donor cells (arrowheads) stratified in a specific band in the outer plexiform layer (OPL) (*). Green: pRPC; Red: synaptophysin. (B). Recoverin (REC) GFP DAPI histology at 12 weeks of survival. It is worth noting that the incidence of GFP/REC‐positive cells was higher in integrated cells as compared to those remaining in the subretinal space (C–J)

The area of GFP‐positive staining was calculated as described in the methods. Examples of engraftment are shown in Figure 4A–C. The effect of cell dose of engraftment is shown as a bar graph in Figure 5A. No GFP‐positive signal was detected in any of the 3 vehicle‐treated eyes taken at 12‐week post‐treatment. In the cell‐treated groups, the positive GFP signal was detected in 81% (21/26) of the cell‐treated eyes. Although not statistically significant, there appeared to be a trend between the area of pRPC engraftment and the dose of cells that was administered. A thickness of 1–3 cell layers was spanned by the GFP‐positive signal. Compared with the 3‐week interval, the GFP‐positive cells were found in greater numbers in the deeper layers in the neural retina at 12 weeks. Generally, although a large number of cells were present in the subretinal space at 12 weeks, some of the implanted cells appeared to have migrated into the host photoreceptor layer up to a depth of 2–3 layers (Figure 4C–E). No GFP‐positive cells were noted in the untreated right eyes.

### 3.5 Transplanted cells integrate and express recoverin, rhodopsin and synaptophysin staining

#### 3.5.1 Synaptophysin

Widespread positive staining of this marker was observed near GFP‐positive pRPC terminals throughout the outer plexiform layer (Figure 5B), suggesting the synaptic integration of grafted pRPCs (in the form of developing photoreceptors) with host bipolar neurons.

#### 3.5.2 Recoverin

Most of the grafted RPCs found in the outer nuclear layers co‐expressed recoverin, so a developing photoreceptor fate was indicated (Figure 5C–J).

**FIGURE 6:**
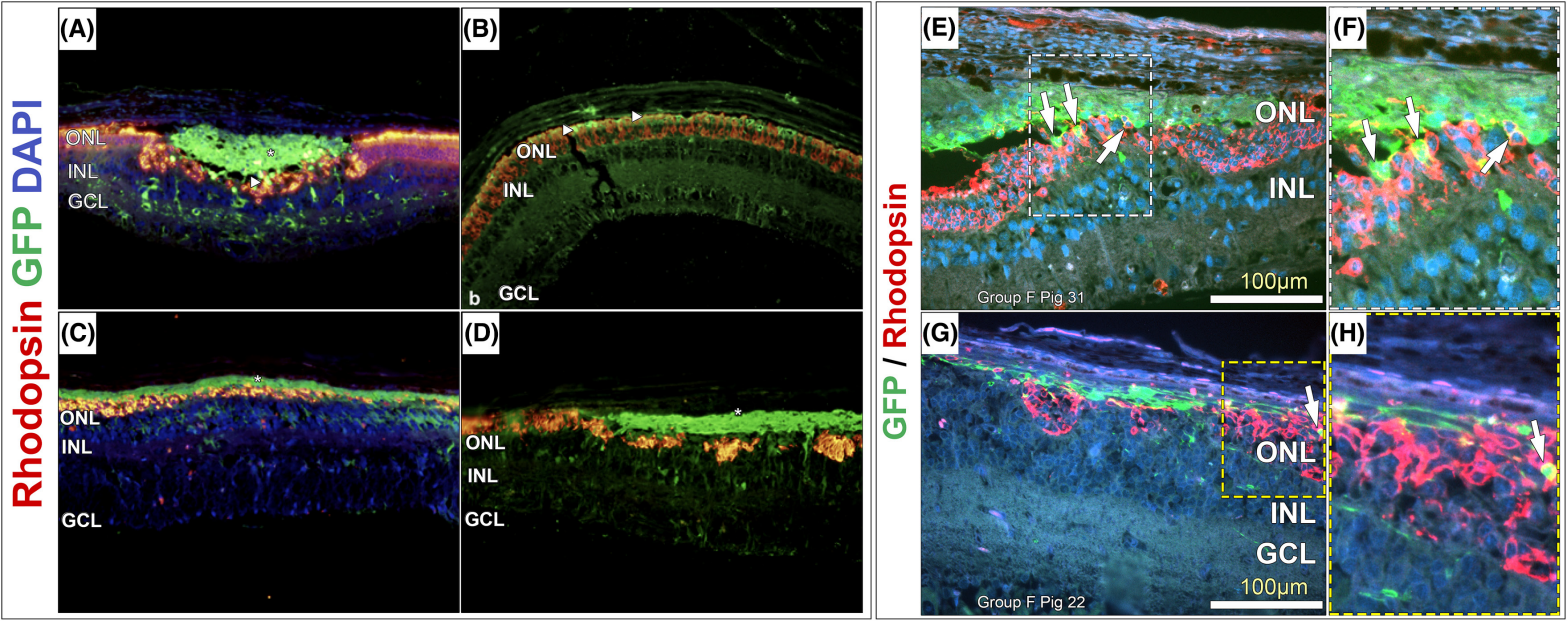
Rhodopsin (RHO) GFP DAPI histology at 12 weeks of survival. In A, a bolus of cells (*) remains mostly undifferentiated in the subretinal space. Several RHO/GFP‐positive cells (arrowhead) could be detected in the ONL. In B, many RHO/GFP‐positive cells (arrowheads) integrated into the photoreceptor layer were detected. In C and D, non‐integrated cells (*) remain negative for RHO. As these cells were also negative for Ki67, it is suggested they remain post‐mitotic retinal progenitor cells and may continue to develop along the retinal photoreceptor lineage after the 12‐week survival time. Higher magnification images showing dual‐labelling of the implanted cells with GFP and rhodopsin (E–H)

Rhodopsin: Only sporadic positive rhodopsin staining that colocalized with GFP was detected, suggesting incomplete differentiation into mature rods at 12 weeks (Figure 6A–H).

A summary of the differentiation of the implanted pRPC is presented in Table 5. Recoverin‐positive and rhodopsin‐positive implanted cells were found to be most prevalent at the higher‐dose groups. It was observed that at 12 weeks after implantation, pRPCs were recoverin‐positive, and rhodopsin‐positive phenotypes were beginning to emerge, particularly in the higher‐dose groups.

**TABLE 5:** Summary of implant differentiation at 12 weeks after implantation

Group	Treatment	Pig ID	Recoverin %	Rhodopsin %
B	0.25 × 10^6^ (50 µl)	15	–	–
E	5 × 10^6^ (100 µl)	7	1.63	–
E	5 × 10^6^ (100 µl)	8	7.25	–
F	10 × 10^6^ (100 µl)	21	2.78	–
F	10 × 10^6^ (100 µl)	22	5.66	7.35
F	10 × 10^6^ (100 µl)	30	4.35	–
F	10 × 10^6^ (100 µl)	31	1.77	1.57
F	10 × 10^6^ (100 µl)	32	1.61	–

### 3.6 Proliferation of survived cells after transplantation

Proliferation data for surviving GFP‐positive cells are presented in Table 6. No cell proliferation was detected in the surviving grafted cells in the subretinal space or the host retina. In the entire study, a single incidence of a proliferating cell was noted in the vitreous.

**TABLE 6:** Proliferation of surviving pRPC at 12 weeks post‐treatment

Group	Treatment	GFP cell counts	Dual‐labelled GFP+Ki67+cells	Mean proliferation index
A	Vehicle	0	0	0
B	0.25 × 10^6^ (50 µl)	59	1	1.7
C	0.25 × 10^6^ (100 µl)	144	0	0
D	1 × 10^6^ (100 µl)	134	0	0
E	5 × 10^6^ (100 µl)	462	0	0
F	10 × 10^6^ (100 µl)	795	0	0

### 3.7 No significant local immune response observed after 12 weeks of transplantation

At 12 weeks, it was found that small numbers of host CD45‐positive cells localized to the injection site were present across treatment groups. No statistically significant effect of treatment on CD45‐positive cells in the retina was revealed by one‐way ANOVA. Local host immune response, as determined using CD45 IHC, is summarized in Table 7.

**TABLE 7:** Host immune response at 12 weeks post‐treatment

Group	Treatment	Mean CD45 cell counts per field (±SEM)
A	Vehicle	0.22 (0.17)
B	0.25 × 10^6^ (50 µl)	0.42 (0.4)
C	0.25 × 10^6^ (100 µl)	1.22 (0.49)
D	1 × 10^6^ (100 µl)	1.96 (1.05)
E	5 × 10^6^ (100 µl)	3.39 (1.83)
F	10 × 10^6^ (100 µl)	4.02 (1.18)

ANOVA *F*_5,28_ = 1.6510, *p* = 0.1866.

### 3.8 Histopathology of major organs demonstrated no findings related to pRPCs

No pRPC‐related histopathology findings were reported in the major organs investigated. The main findings that were recorded were unremarkable and consistent with the type change commonly seen in pigs of this age. All staining controls were stained as expected. No GFP‐positive cells were seen in any non‐target (left‐eye) tissues examined in any study animals.

## 4 DISCUSSION

### 4.1 Surgery procedure and clinical relevance

One primary objective of this study was to develop a protocol for surgery that could be applied to a clinical trial. To that end, verification that the procedures employed were (1) viable in a large animal study and (2) usable in the next clinical application stage, was attempted. It was found that the mini‐pig eye is an excellent surrogate for the human eye. The three‐port pars plana vitrectomy procedure is identical to that used in human surgery and was found to be appropriate and effective for cell delivery to the subretinal space.^17^, ^18^, ^19^ Instrumentation was also investigated and found to be useful and suitable for the intended procedures. Specifically, the cells were safely delivered to the subretinal space by the 38‐gauge cannula. An excellent foundation for developing a clinical protocol for the planned IND is presented in this preclinical study. To overcome the occasional reflux of cells into the vitreous,^20^, ^21^ it is recommended to use a pre‐bleb procedure for greater precision in delivering a single bolus of cells to the subretinal space. As a precautionary measure, it is recommended that topical antibiotics be administered to minimize the risk of post‐surgery infection. We had 7 cases of cataracts out of 39 vitrectomy surgeries, at the long time point (12 weeks). About 18% incidence of cataract in our study. When looking to literature, the incidence of cataracts is very variable and large after vitrectomy surgery and can go from 16% to 66%. The cataracts after vitrectomy are very common, and we stayed on the literature rates. In the real world in multicentre studies of phases 1–3, multiple surgeons and patients of different genders will be involved. So, this study tried to represent this senario.^22^, ^23^, ^24^

### 4.2 Safety of subretinal administration of pRPC

The pig eye is the best model for the study of safety.^25^ Specifically, an allogenic graft best mimics the human clinical trial in a way that xenografts cannot.^26^, ^27^ For example, cell survival throughout the entire study period is optimal, and questions of immunosuppression can be readily addressed (see Section 4.4 below). The procedures employed in this IND‐enabling preclinical study are identical to those proposed by us for clinical use.^28^ Importantly, an excellent safety profile was determined by this 12‐week study. Minimal side effects^29^ and adverse events occurred locally and had no significant effects on the system or major organs. Up to the 12‐week post‐implantation time investigated, no evidence of either significant local or humoral immune response was found. No proliferation of the surviving implanted cells was observed in the subretinal space.

### 4.3 Evidence of cell replacement as a mechanism of action for minimum engrafting dose and recommendation for first‐in‐human (FIH) dose

A minimum engrafting dose of 2.5 × 10^5^ cells is suggested by the pRPC survival data. Although no statistically significant difference between the 50 µl and 100 µl volumes used to deliver this total dose was found, it is recommended that for greater accuracy and practicality, the 100 µl volume is used as the minimum volume for delivery of cells to the subretinal space.

Selected eye sections containing grafted pRPCs were evaluated for GFP co‐expression of the following markers:

1. *Synaptophysin*.^30^ Synaptophysin is considered an integral membrane protein of the synaptic vesicles. Multiple functions in synaptic vesicle formation and exocytosis are served by this marker, including an essential role in neurotransmitter delivery. It is widely used as one of the synaptic function markers and is thought to be closely related to synaptogenesis and synaptic plasticity during neural tissue development. Widespread positive staining of this marker was observed near GFP‐positive pRPC terminals throughout the outer plexiform layer. Therefore, the synaptic integration of grafted pRPCs (in the form of developing photoreceptors) with host bipolar neurons is suggested. Authors agree regarding the material transfer that usually occurs during cell therapy however as it can be seen synaptophysin staining with GFP indicates the cells are able to engraft and make connection with the host tissue.
2. *Recoverin*.^31^ Recoverin is a 23 kilodalton (kDa) neuronal calcium‐binding protein that is primarily detected in the eye's photoreceptor cells. A crucial role in the inhibition of rhodopsin kinase, a molecule that regulates rhodopsin's phosphorylation, is played by this marker. In the context of this study, pRPCs are labelled as developing rod and cone photoreceptors by recoverin. The majority of the grafted RPCs that were found in the outer nuclear layers co‐expressed recoverin, indicating a developing photoreceptor fate.
3. *Rhodopsin*.^32^ Rhodopsin is considered a light‐sensitive receptor protein and a primary biological pigment in the retina's rod photoreceptor cells. It belongs to the G‐protein‐coupled receptor (GPCR) family. Only sporadic positive rhodopsin staining that colocalized with GFP was detected, suggesting incomplete differentiation into mature rods at 12 weeks.

### 4.4 Immunosuppression regimen and recommendation for FIH clinical trial

A previous pilot study by us did not find any effect of immunosuppression on cell survival in normal pigs (RN03‐GE‐0007).^11^ Due to those findings and the immuno‐privileged nature of both the donor cell and the recipient site, cell survival was investigated in an allograft setting that mimics human clinical trials. In this current study, >80% survival at the longest interval (12 weeks) was found at several dose levels, with no evidence of immune rejection either by serum analysis or histopathology.

The primary objective of this study was to investigate the survival and integration of pRPC allotransplants in the mini‐pig retina.^13^ It was determined that cells survived well in all groups, although survival in the 50 µl group was meagre, with only 40% of the animals showing surviving cells at 12 weeks. At 12 weeks, strong evidence of integration was seen, as indicated by the location of the grafted cells within the neuro‐retina, extension of processes to the plexiform layers and expression of key retinal markers such as recoverin and rhodopsin.^14^ Furthermore, survival in allografts without immunosuppression was observed, an informative finding to future clinical work.

The secondary objective was to identify the optimal dosage of implanted cells. A clear relationship between the number of cells injected and found in the retina was seen. An initial dose of 250 k cells in 100 µl is suggested for the first group of Phase I/II safety‐study clinical trials conducted by our group. Less than a twofold larger graft area was produced by a dose of 5 M cells than 1 M cells, so it is suggested that the highest dose for successful engraftment with the highest survival of implanted cells is 1 M cells in 100 µl. However, that the goals of this study were to determine appropriate dosage, method of delivery and anatomical potential of grafted pRPCs. The model we chose was retinal detachment. Although this model shows functional drop in ERG at initiation, this response largely recovers in the following weeks unless the detachment is maintained, an event we sought to avoid to best mirror our clinical work with hRPCs. This does indeed make it very difficult to evaluate the functional potential of grafted cells, and we have therefore avoided any statements regarding photoreceptor function. Not using ERG to check function is a limitation of this study.^33^, ^34^ But is worth noting we are now pursuing further studies in which we will attempt to evaluate retinal and visual function in recipients, but this work was outside the scope of the present study.

In this study, we make use of a heterogenous source of retinal progenitor cells. Although enriched in rod precursor cells, other potentially contaminating cells (of retinal origin) are present in the pRPCs. Other sources such as ES or iPS cells can, through 2d or 3d retinal organoid culture, give rise to RPCs and their progeny rod photoreceptors.^35^ Two main challenges remain to purification of these cells for use therapeutically: (1) dissociation and purification of viable photoreceptor precursors cells; and (2) elimination of pluripotent cells from therapeutic cells. We chose to use foetal donor cells as a source in this study and our clinical work in part to eliminate such challenges in a first‐in‐man study.

## CONFLICT OF INTEREST

M. Abud, P. Baranov, S. Patel, C. Hicks, B. D. Isaac, R. Louzada, M. Ávila, J. Sinden, M. Young: none.

## AUTHOR CONTRIBUTIONS

**Murilo Batista Abud:** Conceptualization (equal); Data curation (equal); Investigation (equal); Methodology (equal); Project administration (equal); Resources (equal); Supervision (equal); Validation (equal); Visualization (equal). **Petr Baranov:** Conceptualization (equal); Data curation (equal); Investigation (equal); Methodology (equal); Project administration (equal); Validation (equal); Visualization (equal); Writing – original draft (equal). **Sara Patel:** Conceptualization (equal); Formal analysis (equal); Methodology (equal); Writing – original draft (equal). **Caroline A. Hicks:** Formal analysis (equal); Resources (equal); Validation (equal). **David Leonardo Cruvinel Isaac:** Data curation (equal); Investigation (equal); Project administration (equal); Supervision (equal); Visualization (equal); Writing – original draft (equal). **Ricardo Noguera Louzada:** Data curation (equal); Investigation (equal); Project administration (equal); Supervision (equal); Visualization (equal); Writing – original draft (equal). **Pierre Dromel:** Data curation (equal); Resources (equal); Validation (equal); Visualization (equal); Writing – original draft (equal). **Deepti Singh:** Data curation (equal); Formal analysis (equal); Project administration (equal); Resources (equal); Software (equal); Validation (equal); Writing – original draft (equal); Writing – review & editing (equal). **John Sinden:** Conceptualization (equal); Funding acquisition (equal); Investigation (equal); Project administration (equal); Supervision (equal); Validation (equal). **Marcos Pereira de Ávila:** Conceptualization (equal); Investigation (equal); Methodology (equal); Project administration (equal); Resources (equal); Supervision (equal); Visualization (equal); Writing – original draft (equal). **Michael Young:** Conceptualization (equal); Data curation (equal); Formal analysis (equal); Funding acquisition (equal); Investigation (equal); Methodology (equal); Project administration (equal); Resources (equal); Supervision (equal); Validation (equal); Visualization (equal); Writing – original draft (equal); Writing – review & editing (equal).

## Data Availability

The data that support the findings of this study are available from the corresponding author upon reasonable request.
